# Statins—Their Effect on Lipoprotein(a) Levels

**DOI:** 10.31083/RCM26162

**Published:** 2025-01-16

**Authors:** Marcin Mateusz Granat

**Affiliations:** ^1^Department of Clinical and Experimental Pharmacology, Faculty of Medicine, Medical University of Warsaw, 02-097 Warsaw, Poland

**Keywords:** lipoprotein(a), statin therapy, cardiovascular disease

## Abstract

Lipoprotein(a) (Lp(a)) serum concentration plays a crucial role as a risk factor in cardiovascular diseases and is gaining more and more attention. Patients with elevated lipoprotein(a) levels are often prescribed statins as they also have high concentrations of low-density lipoprotein cholesterol (LDL-C). Statins are drugs that successfully decrease LDL-C, but their effectiveness in Lp(a) levels reduction is uncertain. The aim of this study was to evaluate if statin therapy can affect Lp(a) concentration. A literature search on databases like PubMed, Oxford Academic, ScienceDirect, Embase, The Cochrane Library, Scopus, and Springer Link was conducted from 1 May to 10 August 2024 with the aim of finding studies concerning the effect of statins on Lp(a) levels. Only randomised control studies and studies with a placebo/comparator arm were included. For calculations, SPSS Statistics software version 29 was used. The risk of bias for this study was assessed using the revised Cochrane risk-of-bias tool for randomised trials. Overall, 43 studies (13,264 participants in study arms and 11,676 in control arms) were included in the analysis. The mean difference of absolute change in Lp(a) concentration for all 43 studies equaled 0.22 mg/dL and was not clinically significant. Egger's regression-based test resulted in no risk of bias in this study (*p* = 0.404). In conclusion, statin therapy does not significantly affect Lp(a) levels. Results of this work suggest that people with high Lp(a) levels will not change their Lp(a)-associated cardiovascular (CV) risk by statin administration.

## 1. Introduction

Lipoprotein(a) (Lp(a)) was discovered in 1963 by Norwegian scientist Kåre Ingmar Berg [1]. It is a particle consisted of cholesterol-rich low-density lipoprotein (LDL) which contains an additional and distinctive plasminogen-like glycoprotein, apolipoprotein(a) (apo(a)), covalently attached to apolipoprotein B. Lp(a) is not affected by lifestyle changes, being genetically determined and dependent on variants of the lipoprotein(a) gene (*LPA* gene) coding for apo(a). Different sizes of apo(a) isoforms have been found, which are determined by the number of kringle IV type 2 (KIV2) structures. The size of apo(a) isoforms correlates inversely with plasma Lp(a) levels because the larger the isoform is, the longer intracellular processing and increased intracellular degradation of it is observed [2, 3].

There is strong evidence linking Lp(a) levels to atherosclerosis and thrombosis [4, 5, 6, 7]. Lp(a) promotes pathophysiological processes via four mechanisms: vascular inflammation, atherogenesis, calcification, and thrombosis. The molecules of Lp(a) enter arterial intima through pores. In this location Lp(a) undergoes oxidation and reactive oxygen species are produced. Subsequently, their presence induces inflammation through cytokine production, vascular wall remodeling, augmented endothelial permeability, and apoptosis. The oxidized Lp(a) is engulfed by macrophages through its scavenger receptor CD36 to generate the foam cells and promote atherosclerotic plaque formation. Lp(a) increases both the production and activity, of tissue-type plasminogen activator inhibitor-1, which leads to a decrease in fibrinolysis, finally ending in thrombosis [4, 8]. All these processes escalate in persons with high Lp(a) levels, increasing their risk of cardiovascular disease (CVD) development.

CVDs such as coronary heart disease are nowadays the most common cause associated with human mortality worldwide [9]. Among many factors that increase the risk of CVD development is elevated levels of Lp(a) [10]. The serum concentration of Lp(a) is currently gaining more and more clinical significance and the Lp(a) research field is under constant development [2]. It is of the utmost importance to underscore the independence of Lp(a) from traditional risk factors of CVDs, like, e.g., high blood pressure, diabetes, hypercholesterolaemia, smoking tobacco products, obesity or being overweight, inactivity, and family history of CVDs [11]. Nowadays, there are numerous publications describing Lp(a) levels as an independent risk factor corresponding to the mortality rate in CVDs [12, 13, 14, 15, 16, 17]. An extensive analysis on Lp(a) levels as the risk factor of CVDs was published by Ghose [16]. In this work, the author includes large observational studies, Mendelian randomization studies, genome wide variation studies, meta-analyses, and interventional trials. His work surmises that between highest and lowest Lp(a), there is an increased risk of myocardial infarction (3–4 fold), valvular aortic stenosis (3 fold), coronary artery stenosis (5 fold), carotid stenosis (1.7 fold), ischemic stroke (1.6 fold), heart failure (1.5–2 fold), and cardiovascular mortality (1.5 fold) [16]. Erqou *et al*. [17] examined 24 cohort studies finding continuous and independent associations of Lp(a) levels with coronary heart disease morbidity. A large study including 27,756 individuals was published by Wong *et al*. [15]. In this pooled and multi-ethnic cohort, researchers proved that Lp(a) concentration is correlative with increased atherosclerotic cardiovascular disease (ASCVD) risk (hazard ratios for ASCVD events and Lp(a) levels in the 50th to <75th, 75th to <90th, and ≥90th percentiles compared with Lp(a) levels <50th percentile were respectively 1.06 (95% confidence interval (CI): 0.99–1.14), 1.18 (95% CI: 1.09–1.28), and 1.46 (95% CI: 1.33–1.59)) [15]. Langsted *et al*. [12] studied approximately 100,000 people from the Copenhagen General Population Study and observed that the hazard ratio of myocardial infarction with elevated Lp(a) levels was 1.6 (1.4–1.9). What is more, a 15 mg/dL increase in Lp(a) was associated with a hazard ratio for CV mortality of 1.18 (1.12–1.25) [12]. Kronenberg [13] stated that Lp(a) is one of the strongest genetically determined risk factors for CVDs. He pointed out that the Copenhagen City Heart Study observed for individuals from a general population with concentrations between 30 and 76 mg/dL had a 1.6 fold increased risk for incident myocardial infarction compared to persons with Lp(a) levels below 5 mg/dL. The risk increased to 1.9 for individuals with Lp(a) concentrations between 77 and 117 mg/dL and to 2.6 for those with Lp(a) concentrations above 117 mg/dL [13]. Recently, a systematic review and meta-analysis was published by Tian *et al*. [14] with conclusions that after pooling 49 studies, the association between elevated Lp(a) levels and overall ASCVD is significant (*p* < 0.001).

The central role of Lp(a) in CVDs is reflected in modern guidelines consensus statements published by e.g., the American Heart Association (AHA), Canadian Cardiovascular Society (CCS), Chinese Society of Cardiology, and Polish Lipid Association. AHA guidelines set the Lp(a) threshold at >50 mg/dL, while CCS proposes ≥50 mg/dL. Chinese Society of Cardiology sets the cut-off for Lp(a) levels at 30 mg/dL, while the Polish Lipid Association defines CV risk according to specific a Lp(a) serum concentration range: moderate CV risk (30–50 mg/dL), high CV risk (>50 mg/dL), and very high CV risk (>180 mg/dL) [18, 19, 20, 21]. It is worth mentioning that changes to Lp(a) levels between men and women are marginal, but significant differences are observed in patients of variant ethnicity. The highest median Lp(a) levels are among Blacks, who are followed by South Asians, Whites, and Chinese [6].

Both the association of elevated Lp(a) levels with increased risk of CVDs and mortality as well as the guidelines’ recommendations to maintain Lp(a) concentration at a desirable level, prompted clinicians to seek pharmacological agents that effectively decrease Lp(a) concentration. To achieve this goal, statins were brought to attention as they are an important group of drugs that reduce CV risk. Statins were discovered in 1966 by Japanese researchers, Akira Endo and his co-workers, during experiments on mould fungi [22]. Currently, they are widely used cholesterol-lowering medicines that act mainly by inhibiting 3-hydroxy-3-methyl-glutaryl-coenzyme A (HMG-CoA) reductase, the enzyme activity of which, is crucial to cholesterol and other sterols biosynthesis. All statins successfully reduce low-density lipoprotein cholesterol (LDL-C), but the extent of that effect depends on the statin and its dose. Moreover, they reduce triglyceride levels and slightly increase high-density lipoprotein cholesterol (HDL-C). Statins are also characterised by pleiotropic effects, which have favourable effects such as improving endothelial function, suppressing T-cell lymphocytes, decreasing platelet aggregation, and reducing antioxidant activity caused by different mechanisms. Their cardioprotective properties and broad impact on lipid profile make statins frequently prescribed medicines worldwide [22]. However, the impact of statins on Lp(a) levels remains uncertain, necessitating a systematic review and analysis. The aim of this study was to determine whether statins have an effect on Lp(a) levels based on the literature search. Providing an answer to this problem would be beneficial as it would bring to light information on if this commonly used group of drugs may be useful to treat patients with elevated Lp(a) levels.

## 2. Materials and Methods

A comprehensive systematic search on databases like PubMed, Oxford Academic, ScienceDirect, Embase, The Cochrane Library, Scopus, and Springer Link was conducted from 1 May to 10 August 2024. The choice of the aforementioned databases was made based on their leading role in scientific research and English-language content. In the search on PubMed (accessed from 16 to 31 July 2024), Oxford Academic (accessed from 1 to 10 August 2024), ScienceDirect (accessed from 31 May to 14 June 2024), The Cochrane Library (accessed from 15 to 30 June 2024), and Springer Link (accessed from 1 to 15 July 2024) the phrases “statin lipoprotein(a)”, “statin Lp(a)”, and “statin lipoprotein effect” were used. Because Embase and Scopus browsers do not support round brackets signs, the search in those databases included the phrases: “statin lipoprotein a”, “statin Lp a”, and “statin lipoprotein effect”. The Embase database was accessed from 1 to 15 May 2024 and Scopus from 16 to 30 May 2024. The publications considered in the analysis involved the whole time-span available in the databases up to 10 August 2024. The following inclusion criteria were selected: statin therapies with measured effect on Lp(a) concentration, randomised controlled trials (RCTs), and studies with both, a statin arm and control arm (placebo or comparator). The exclusion criteria included: studies that lasted shorter than 3 weeks, cerivastatin studies, editorial comments, meta-analyses, duplicates, and studies with no control group.

Study selection was performed by the undermentioned steps. Firstly, with the use of the above phrases, studies on the subject of the effect of statins on Lp(a) levels was searched. The eligibility of these studies was based on their title and abstract. After that, duplicates and meta-analyses were excluded. Subsequently, extensive full-text research on articles was performed to include only studies that met the criteria. Cerivastatin studies were excluded at this stage as it was withdrawn from the market due to unacceptable adverse effects [23]. The final step divided studies into three groups: with the results reporting that statins either statistically significantly increase, reduce, or have no effect on the Lp(a) levels. To analyse the overall effect of statins on Lp(a) levels, weighted mean differences and the mean difference of absolute change (MDAC) in Lp(a) concentrations were calculated.

The risk of bias for this study was assessed using the revised Cochrane risk-of-bias tool for randomised trials [24]. The evaluation of the five following domains was conducted: randomization procedure, measurement of the outcome, missing outcome data, deviations from intended interventions, and selection of the presented result. To detect publication bias, Egger’s regression-based test was performed. For all calculations, SPSS Statistics software version 29 (IBM, Armonk, New York, NY, USA) was used and a 95% confidence interval of the difference was applied so *p* < 0.05 was considered statistically significant.

## 3. Results

**Fig. 1.**
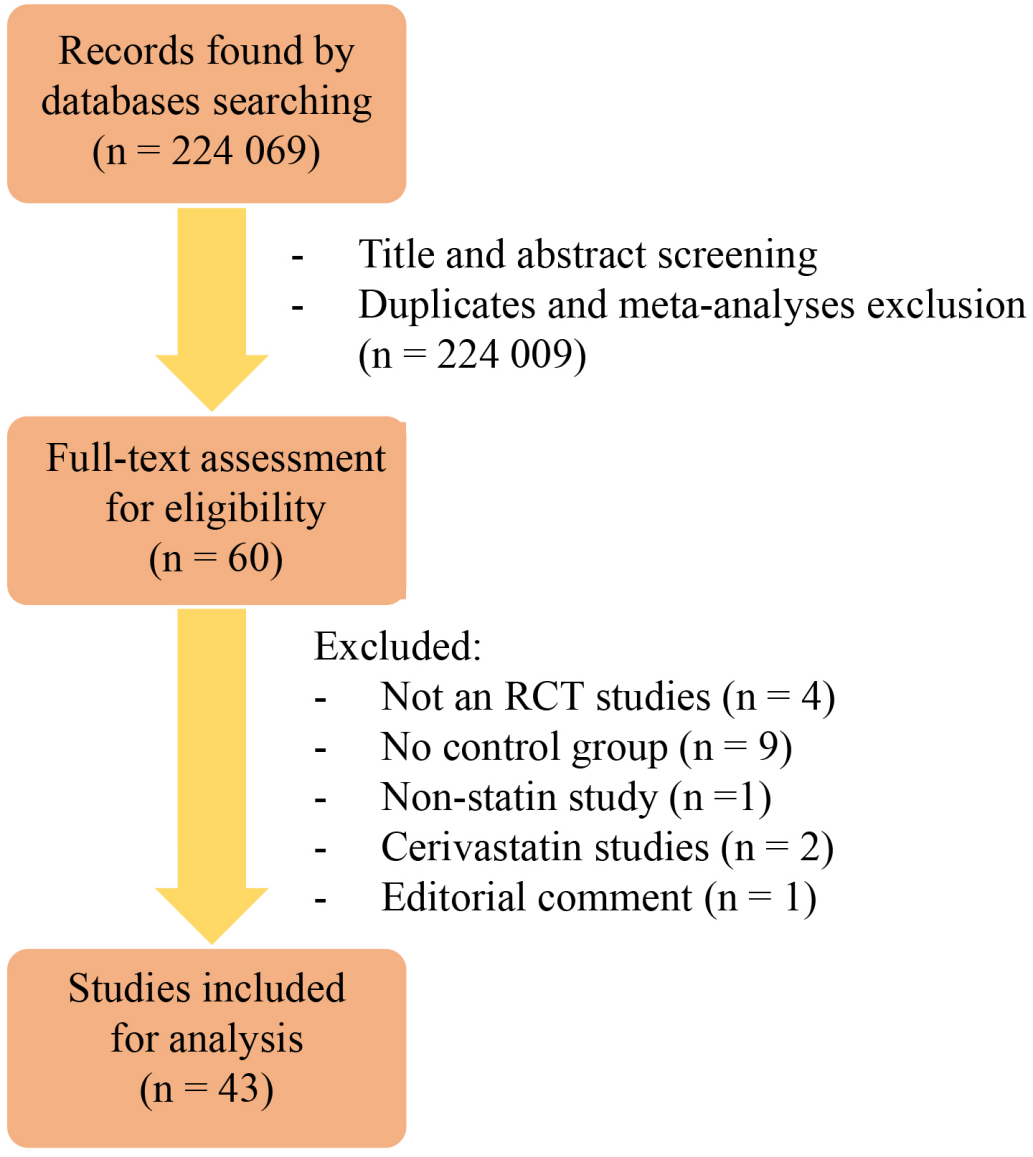
**The scheme presenting the process of studies inclusion**. Abbreviations: RCT, randomised controlled trial.

Initially, this study yielded 224,069 records. After screening the titles and abstracts as well as excluding duplicates and meta-analyses, 60 records remained. The full-text assessment for these studies resulted in a further rejection of 17 records as they did not meet the criteria of this research. Finally, 43 studies were included for further analysis (see Fig. 1).

Those records were subsequently divided into three groups:

∙ Group 1 – studies resulted in a statistically significant increase of Lp(a) levels (n = 4) (see Table 1, Ref. [25, 26, 27, 28]).

∙ Group 2 – studies resulted in a statistically significant reduction of Lp(a) levels (n = 4) (see Table 2, Ref. [29, 30, 31, 32]).

∙ Group 3 – studies with the results reporting that statins have no statistically significant effect on Lp(a) levels (n = 35) (see Table 3, Ref. [33, 34, 35, 36, 37, 38, 39, 40, 41, 42, 43, 44, 45, 46, 47, 48, 49, 50, 51, 52, 53, 54, 55, 56, 57, 58, 59, 60, 61, 62, 63, 64, 65, 66, 67]).

**Table 1.** **Characteristics of the 4 studies from group 1**.

Study	Type of statin (daily dosage)	Duration of the study	Study arm	Control arm
Capoulade *et al*., 2015 [25]	Rosuvastatin (40 mg)	1 year	n = 112	n = 108, placebo
Khera *et al*., 2014 [26]	Rosuvastatin (20 mg)	12 months	n = 3882	n = 3862, placebo
Ky *et al*., 2008 [27]	Atorvastatin (10 mg and 80 mg), pravastatin (40 mg)	16 weeks	n = 29 (atorvastatin 10 mg)	n = 27, placebo
			n = 26 (atorvastatin 80 mg)	
			n = 24 (pravastatin 40 mg)	
Rodenburg *et al*., 2006 [28]	Pravastatin (40 mg)	2 years	n = 90	n = 88, placebo

**Table 2.** **Characteristics of the 4 studies from group 2**.

Study	Type of statin (daily dosage)	Duration of the study	Study arm	Control arm
Ballantyne *et al*., 2003 [29]	Atorvastatin (10, 20, 40, and 80 mg)	12 weeks	n = 248	n = 60, placebo
Dallongeville* et al*., 1994 [30]	Fluvastatin (2.5, 5, 10, and 20 mg)	6 weeks	n = 80 (fluvastatin 2.5 mg)	n = 80, placebo
			n = 82 (fluvastatin 5 mg)	
			n = 82 (fluvastatin 10 mg)	
			n = 83 (fluvastatin 20 mg)	
Hernández *et al*., 2011 [31]	Atorvastatin (10 and 40 mg)	3 months	n = 21 (atorvastatin 10 mg)	n = 19, placebo
			n = 22 (atorvastatin 40 mg)	
Schaefer *et al*., 2002 [32]	Atorvastatin (20, 40, and 80 mg)	24 weeks	n = 103	n = 88, placebo

**Table 3.** **Characteristics of the 35 studies from group 3**.

Study	Type of statin (daily dosage)	Duration of the study	Study arm	Control arm
Athyros *et al*., 2002 [33]	Atorvastatin (20 mg)	24 weeks	n = 40	n = 40, 200-mg micronized fenofibrate
Avellone *et al*., 1994 [34]	Pravastatin (20 mg)	24 weeks	n = 10	n = 10, placebo
Bevilacqua *et al*., 1997 [35]	Fluvastatin (40 mg)	20 weeks	n = 25	n = 23, placebo
Blann *et al*., 2001 [36]	Pravastatin (40 mg)	4 months	n = 17	n = 15, placebo
Broyles *et al*., 1995 [37]	Fluvastatin (20 mg)	6 weeks	n = 37	n = 20, placebo
Crouse *et al*., 1995 [38]	Pravastatin (10, 20, and 40 mg)	24 months	n = 64	n = 70, placebo
Canas *et al*., 2015 [39]	Atorvastatin (10 and 20 mg)	6 months	n = 19	n = 19, placebo
Cobbaert *et al*., 1992 [40]	Pravastatin (10 and 20 mg)	12 weeks	n = 47	n = 50, placebo
Cobbaert *et al*., 1997 [41]	Pravastatin (40 mg)	24 months	n = 358	n = 346, placebo
Davidson *et al*., 2002 [42]	Simvastatin (10, 20, 40, or 80 mg)	12 weeks	n = 261	n = 69, placebo
Dujovne *et al*., 2001 [43]	Lovastatin (40 mg)	8 weeks	n = 25	n = 24, placebo
Dupuis *et al*., 1999 [44]	Pravastatin (40 mg)	6 weeks	n = 28	n = 27, placebo
Goldberg *et al*., 2004 [45]	Simvastatin (10, 20, 40, or 80 mg)	12 weeks	n = 199	n = 51, placebo
Haffner *et al*., 1995 [46]	Simvastatin (10 and 20 mg)	24 weeks	n = 80 (simvastatin 10 mg)	n = 82, placebo
			n = 166 (simvastatin 20 mg)	
Hunninghake *et al*., 1993 [47]	Pravastatin (10, 20, and 40 mg)	12 weeks	n = 79	n = 46, placebo
Insull *et al*., 2005 [48]	Simvastatin (10 mg)	12 weeks	n = 25	n = 23, placebo
Kerzner *et al*., 2003 [49]	Lovastatin (12, 20, and 40 mg)	12 weeks	n = 220	n = 64, placebo
Kollerits *et al*., 2016 [50]	Atorvastatin (10 and 20 mg)	6 months	n = 603	n = 630, placebo
Kostis *et al*., 1994 [51]	Pravastatin (40 mg), lovastatin (40 mg)	6 weeks	n = 17 (pravastatin)	n = 17, placebo
			n = 17 (lovastatin)	
Lepre *et al*., 1999 [52]	Simvastatin (5 and 10 mg)	12 weeks	n = 32	n = 17, placebo
Melani *et al*., 2003 [53]	Pravastatin (10, 20, and 40 mg)	12 weeks	n = 205	n = 65, placebo
Min *et al*., 2013 [54]	Atorvastatin (20 mg)	4 weeks	n = 43	n = 46, placebo
Mishra *et al*., 2005 [55]	Atorvastatin (10 mg)	12 weeks	n = 11	n = 11, placebo
Nawrocki *et al*., 1995 [56]	Atorvastatin (2.5, 5, 10, 20, 40, and 80 mg)	6 weeks	n = 11 (atorvastatin 2.5 mg)	n = 12, placebo
			n = 13 (atorvastatin 5 mg)	
			n = 11 (atorvastatin 10 mg)	
			n = 10 (atorvastatin 20 mg)	
			n = 11 (atorvastatin 40 mg)	
			n = 11 (atorvastatin 80 mg)	
Nestel *et al*., 2013 [57]	Pravastatin (40 mg)	1 year	n = 3941	n = 3922, placebo
Nielsen *et al*., 1993 [58]	Simvastatin (10 and 20 mg)	18 weeks	n = 8	n = 10, placebo
Notarbartolo *et al*., 1995 [59]	Simvastatin (20 mg)	3 months	n = 12	n = 11, placebo
Saltissi *et al*., 2002 [60]	Simvastatin (5 mg)	24 weeks	n = 12 (hemodialysis)	n = 12, placebo
			n = 10 (peritoneal dialysis)	
Schanberg *et al*., 2012 [61]	Atorvastatin^a^ (10 or 20 mg)	3 years	n = 113	n = 108, placebo
Schrott *et al*., 1995 [62]	Lovastatin (40 mg)	12 weeks	n = 24	n = 24, placebo
Stein *et al*., 2000 [63]	Simvastatin (40 and 80 mg)	6 weeks	n = 127 (simvastatin 40 mg)	n = 124, placebo
			n = 127 (simvastatin 80 mg)	
Tsimikas *et al*., 2004 [64]	Atorvastatin (80 mg)	16 weeks	n = 1151	n = 1190, placebo
Wiegman *et al*., 2004 [65]	Pravastatin (20 and 40 mg)	2 years	n = 106	n = 107, placebo
Winkler *et al*., 2004 [66]	Fluvastatin (80 mg)	8 weeks	n = 42	n = 47, placebo
Zambon *et al*., 1994 [67]	Pravastatin (20 mg)	16 weeks	n = 12	n = 12, placebo

^a^Dose was 10 or 20 mg depending on the participant’s age and weight.

The duration of the analysed studies ranged from 4 weeks to 3 years. The number of participants also varied. In the group of 4 studies representing data indicating that statins significantly increase Lp(a) levels, the study arm included 4163 subjects and the control arm of 4085 individuals. Group 2 with the results of significant reduction of Lp(a) levels also consisted of 4 studies, but the aggregate number of participants was smaller: the study arm equalled 721 and the control arm equalled 247. The largest group was composed of 35 studies with the data informing that statins do not affect Lp(a) levels in statistically significant way. There were 8380 participants in the study arm, and 7344 in the control arm. What is more, in this group the biggest individual study by Nestel *et al*. [57] was present with the study arm of 3941 participants and the placebo arm of 3922.

In total, six different statins were investigated: atorvastatin (12 studies), fluvastatin (4 studies), lovastatin (4 studies), pravastatin (14 studies), rosuvastatin (2 studies), and simvastatin (9 studies). In group 1, atorvastatin (1 study), pravastatin (2 studies), and rosuvastatin (2 studies) were present. Group 2 included studies concerning only two statins: atorvastatin (3 studies) and fluvastatin (1 study). In group 3, five types of statins were found: atorvastatin (8 studies), fluvastatin (3 studies), lovastatin (4 studies), pravastatin (12 studies), and simvastatin (9 studies).

All studies were analysed concerning participants age, sex, and baseline Lp(a) levels. Most of the studies [25, 26, 27, 28, 29, 31, 32, 33, 35, 36, 38, 39, 41, 42, 43, 44, 46, 47, 48, 49, 50, 52, 53, 54, 55, 56, 57, 58, 59, 60, 61, 62, 63, 64, 65, 66, 67] included information about the age of participants and only three of them [28, 61, 65] conducted research on juveniles with an overall mean age of 14 years. In the adult studies, the overall mean age equalled 60 years, with the lowest mean age of 48 years presented in the work by Notarbartolo *et al*. [59] and the highest mean age of 67 years in the research by Winkler *et al*. [66]. In many studies [25, 26, 27, 28, 29, 31, 32, 33, 35, 36, 38, 39, 42, 43, 44, 45, 46, 47, 49, 50, 51, 52, 53, 54, 55, 56, 57, 58, 59, 60, 61, 62, 63, 64, 66, 67] the sex of the participants was described which resulted in the conclusion that on average 61% of participants were males and 39% were females. Moreover, numerous studies presented baseline levels of Lp(a) in subjects [25, 26, 27, 28, 31, 33, 34, 35, 37, 38, 42, 44, 45, 46, 47, 48, 49, 51, 52, 54, 57, 58, 59, 60, 62, 63, 64, 65, 66, 67]. The lowest mean value of 7.8 mg/dL was reported in the work by Rodenburg *et al*. [28] and the highest mean value of 89.1 mg/dL was included in the study by Capoulade *et al*. [25]. While the overall mean value of baseline Lp(a) concentration in all studies equaled 25.8 mg/dL, only one study by Capoulade *et al*. [25] separated participants into two groups: those with low (≤58.5 mg/dL) and high (>58.5 mg/dL) baseline Lp(a).

To provide an answer if statin therapy affects Lp(a) concentration, calculations of weighted mean differences and MDAC were performed. MDAC of Lp(a) levels in the statin vs. the control arms in all 43 studies equalled 0.22 mg/dL. As studies included in this work concerned different statins, MDAC for subgroups created by statin type were calculated as well, and the results were as follows: atorvastatin studies (MDAC = –2.72 mg/dL), fluvastatin studies (MDAC = 0.77 mg/dL), lovastatin studies (MDAC = –0.05 mg/dL), pravastatin studies (MDAC = 0.20 mg/dL), rosuvastatin studies (MDAC = 3.90 mg/dL), and simvastatin studies (MDAC = –1.04 mg/dL). By comparison of the number of studies in each of the three groups, using the participant population in those studies and MDAC calculations, the finding of this analysis states that statin therapy does not cause clinically important changes in Lp(a) levels.

Results of the risk of bias for this work are presented in **Supplementary Table 1**. None of the studies were evaluated as having a low risk of overall bias. The majority of studies were double-blinded. Six studies either did not provide information on blinding or were open-label trials [30, 32, 33, 38, 40, 63]. The risk of bias due to the measurement of outcomes and missing outcome data was low in all studies. **Supplementary Fig. 1** shows the funnel plot of mean difference changes. According to visual inspection as well as Egger’s regression-based test, there was no indication of publication bias in this study (*p* = 0.404).

## 4. Discussion

Contemporarily, the mechanism behind the effect of statins on Lp(a) levels is unknown. However, it has been hypothesised that Lp(a), despite its structural similarity to LDL-C, may be cleared from plasma through another pathway. LDL receptors take part in LDL-C plasma clearing and statins upregulate them. Nevertheless, Lp(a) concentration during statin therapy remains unaffected which leads to the conclusion that decreasing Lp(a) levels may need a different target than LDL receptors and may be associated with Lp(a) metabolism [68]. The answer to this may be delivered by further research providing greater knowledge on mechanisms of Lp(a) synthesis and actions, eventually leading to novel therapeutic agents [69]. The fact that the *LPA* gene coding for apo(a) is characterised by polymorphism is also worth noting. While published studies generally lack consideration of Lp(a) genetic regulators, they may play a crucial role in Lp(a) metabolism and changes of its levels in response to pharmacotherapy [70].

To the best of my knowledge, the latest comprehensive meta-analysis focused on the impact of statins on Lp(a), was presented by de Boer *at al*. [23] and the conclusions of that study were the same as is stated here. I decided to conduct new research to assess possible new studies as de Boer *et al*. [23] completed a search up until August 2019. What is more, de Boer *at al*. [23] only included RCT studies with the study arm and placebo arm, while in this search I also included RCTs with the study arm and comparator arm. Knowledge of statins and their effect on Lp(a) is still evolving [71]. In 2020, Tsimikas* et al*. [72] published an analysis with the same aim as this work showing a significant increase in Lp(a) levels following statin treatment. Earlier valuable research concerning this topic was written by Takagi and Umemoto [73]. Although their work focused on atorvastatin alone, it included an analysis of nine trials and the conclusion of a significant decrease of Lp(a) levels in the study arm compared to the placebo arm.

While my study search covered up until 10 August 2024, it would be beneficial to conduct future analyses centred around statin treatment and Lp(a) levels. It may be valuable to plan research that would assess the effectiveness of statins on patients with high baseline Lp(a) concentrations (e.g., >50 mg/dL) outside the range of 10–35 mg/dL, as was the case in most studies analysed in this work. Broadening the range of subjects’ age, especially to adolescents and young adults as well as including patients above 70 years old may also contribute to useful results. Finally, as the physiological function of Lp(a) is still not clear [74], it would be of great importance to extensively study this particle from the moment of its formation to its degradation. Since we know that oxidized Lp(a) binds to macrophages, which is one of the steps leading to pathological changes, developing agents stopping this process may be beneficial. Inventing and testing substances that would act intracellularly and reduce Lp(a) biosynthesis can be beneficial too, as a similar mechanism proved effective in statins and decreased LDL-C. Although Lp(a) is correlated with platelet aggregation, further investigation is needed to acquire full knowledge of this exact mechanism [74]. Future studies comprehensively explaining this subject may give a chance for the development of new medical substances that would not affect Lp(a) concentration, but rather attenuate its pathological impact on thrombocytes.

Given the finding that statins may lack the ability to reduce Lp(a) concentrations, a search for alternative therapeutic options may be required. The most effective and clinically available Lp(a)-lowering method is a lipoprotein apheresis which results in a reduction of mean interval Lp(a) of 25–40% [75]. Long-term studies on lipoprotein apheresis state that it may reduce 5-year CV risk by up to 86% [76]. Among pharmacologic agents, proprotein convertase subtilisin/kexin type 9 (PCSK9) inhibitors are known for an approximate 20–30% reduction in Lp(a) levels and attenuation of arterial wall inflammation caused by high Lp(a) [75, 77]. Monoclonal antibodies, alirocumab and evolocumab, target the proteolytic enzyme PCSK9 which plays a role in regulating lipid serum concentration [78]. Another interesting drug is inclisiran, a small interfering ribonucleic acid (siRNA) which targets intracellular PCSK9. It has been demonstrated that inclisiran can reduce Lp(a) levels by approximately 20% [79]. Lomitapide is also worth mentioning. This is a drug indicated for patients with homozygous familial hypercholesterolemia, but according to the study by Cuchel *et al*. [80], it is also effective in reducing Lp(a) levels by 15–19%. Although the Lp(a)-lowering mechanism of lomitapide is unclear, it may be associated with decreasing levels of very low-density lipoprotein and chylomicron synthesis via inhibition of microsomal triglyceride transfer protein [75].

## 5. Conclusions

All four studies [25, 26, 27, 28] from group 1 and all four studies [29, 30, 31, 32] from group 2 concluded that statin treatment affects Lp(a), respectively by increasing or reducing its levels. The number of these studies is low compared to 35 studies within group 3. Calculations of weighted mean difference and MDAC of all 43 studies as well as studies separated into subgroups by statin type provide information that statins do not significantly affect Lp(a) concentration.

As previously mentioned, many studies presented data on patients’ baseline Lp(a) concentration. However, it is worth noting that the diversity of baseline Lp(a) among participants was relatively small: in 22 out of 30 studies, its concentration was within the range of 10–35 mg/dL. The same conclusion can be drawn regarding the participants’ age. Among 38 studies, only 3 focused on juveniles and 30 conducted research with the participation of people within the age range of 50 to 65. Unfortunately, this made it impossible for this study to examine the effect of statins on populations with various baseline Lp(a) concentrations and age. Large numbers of male and female participants were present in all studies, so determining the effectiveness of statins separately for both sexes would have been interesting, but could not be achieved as none of the authors stratified their results based on sex.

It is worth noticing that group 1 had its limitations. One of these limitations is related to the risk of bias. It is so because the overall outcomes of 3/4 studies in this group were evaluated with a high risk of bias. Therefore, the percentage of studies with a high risk of bias over the overall outcome in this group is the highest and equals 75% (see **Supplementary Table 1**). What is more, Capoulade *et al*. [25] stated that Lp(a) was measured in stored serum and post hoc analysis was performed, so the results should be interpreted with caution. The rosuvastatin effect on Lp(a) levels is also problematic in work by Khera *et al*. [26]. It is true, that this statin resulted in a statistically significant positive shift in the overall Lp(a) distribution (*p* < 0.0001), but median change in Lp(a) with rosuvastatin and placebo was zero. Another shortcoming is present in the study by Rodenburg *et al*. [28]. It is of the highest importance to note, that the population study in this research is very similar to the population present in the research by Wiegman *et al*. [65] which is stated by the authors in the methods section. What is more, the authors declare performing a double-blind randomised placebo-controlled trial, but the randomization procedure is not described at all.

The shortcomings of group 2 were also noticeable. First of all, group 2 presented the smallest number of all participants (968 participants) compared to the other groups (8248 participants in group 1 and 15,724 participants in group 3). It is important to note that all studies in group 2 were randomised, however, the exact algorithm of this process was not explained in depth. Additionally, Ballantyne *et al*. [29] and Hernández *et al*. [31] had an extensive exclusion criteria in their work which may prevent the extrapolation of the results to other populations. It must also be noted that Dallongeville* et al*. [30] conducted the study for an extremely short period of 6 weeks, which makes analysis of the long-term efficacy, problematic.

## Abbreviations

AHA, American Heart Association; apo(a), apolipoprotein(a); ASCVD, atherosclerotic cardiovascular disease; CCS, Canadian Cardiovascular Society; CI, confidence interval; CV, cardiovascular; CVDs, cardiovascular diseases; HDL-C, high-density lipoprotein cholesterol; HMG-CoA, 3-hydroxy-3-methyl-glutaryl-coenzyme A; KIV2, kringle IV type 2; *LPA *gene, lipoprotein(a) gene; LDL, low-density lipoprotein; LDL-C, low-density lipoprotein cholesterol; Lp(a), lipoprotein(a); MDAC, mean difference of absolute change; RCTs, randomised controlled trials; PCSK9, proprotein convertase subtilisin/kexin type 9; siRNA, small interfering ribonucleic acid.

## Supplementary Material

Supplementary material associated with this article can be found, in the online version, at https://doi.org/10.31083/RCM26162.
