# Supplementary material for: Statins—Their Effect on Lipoprotein(a) Levels
Source: Rev Cardiovasc Med. 2025 Jan 16;26(1):26162. doi: 10.31083/RCM26162 (PMC11760552; doi:10.31083/RCM26162)

**Supplementary Fig. 1**. Funnel plot for effect estimated in meta-analysis of absolute change in Lp(a) levels.


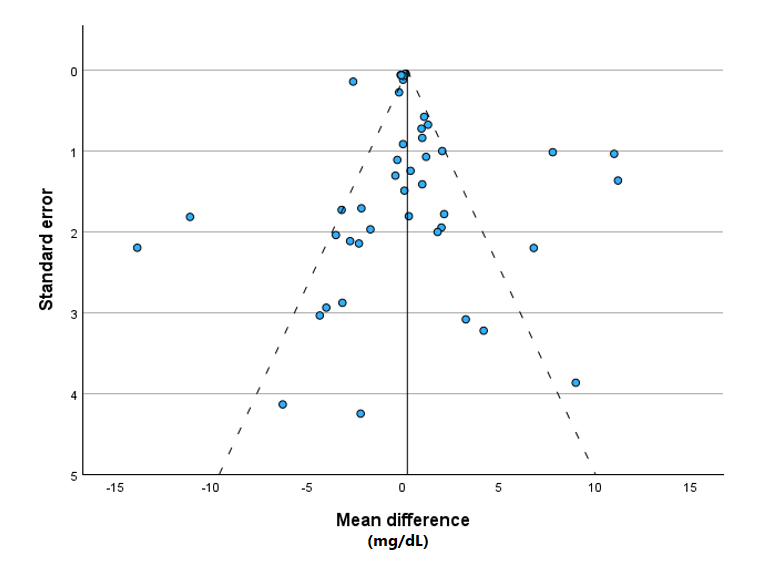

Supplement: Supplementary file 1 [file 2153-8174-26-1-26162-s1.zip › Supplementary Fig. 1.docx]
