# Supplementary material for: Statins—Their Effect on Lipoprotein(a) Levels
Source: Rev Cardiovasc Med. 2025 Jan 16;26(1):26162. doi: 10.31083/RCM26162 (PMC11760552; doi:10.31083/RCM26162)
Supplement: Supplementary file 1 [file 2153-8174-26-1-26162-s1.zip › Supplementary Table 1.docx]

**Supplementary Table 1**. Risk of bias table for all studies. ✓ - low risk, ⮃ - some concerns, ! – high risk.

| Study | Randomization procedure | Measurement of the outcome | Missing outcome data | Deviations from intended interventions | Selection of the presented result | Overall outcome |
| --- | --- | --- | --- | --- | --- | --- |
| Capoulade, 2015 | ✓ | ✓ | ✓ | ! | ⮃ | ! |
| Khera, 2014 | ✓ | ✓ | ✓ | ! | ⮃ | ! |
| Ky, 2008 | ⮃ | ✓ | ✓ | ! | ⮃ | ! |
| Rodenburg, 2006 | ⮃ | ✓ | ✓ | ✓ | ⮃ | ⮃ |
| Ballantyne, 2003 | ⮃ | ✓ | ✓ | ✓ | ⮃ | ⮃ |
| Dallongeville, 1994 | ⮃ | ✓ | ✓ | ✓ | ⮃ | ⮃ |
| Hernández, 2011 | ⮃ | ✓ | ✓ | ⮃ | ⮃ | ⮃ |
| Schaefer, 2002 | ⮃ | ✓ | ✓ | ✓ | ⮃ | ⮃ |
| Athyros, 2002 | ✓ | ✓ | ✓ | ⮃ | ⮃ | ⮃ |
| Avellone, 1994 | ⮃ | ✓ | ✓ | ! | ⮃ | ! |
| Bevilacqua, 1997 | ⮃ | ✓ | ✓ | ✓ | ⮃ | ⮃ |
| Blann, 2001 | ⮃ | ✓ | ✓ | ✓ | ⮃ | ⮃ |
| Broyles, 1995 | ! | ✓ | ✓ | ✓ | ⮃ | ! |
| Crouse, 1995 | ⮃ | ✓ | ✓ | ! | ⮃ | ! |
| Canas, 2015 | ⮃ | ✓ | ✓ | ✓ | ⮃ | ⮃ |
| Cobbaert, 1992 | ⮃ | ✓ | ✓ | ⮃ | ⮃ | ⮃ |
| Cobbaert, 1997 | ⮃ | ✓ | ✓ | ! | ⮃ | ! |
| Davidson, 2002 | ✓ | ✓ | ✓ | ✓ | ⮃ | ⮃ |
| Dupuis, 1999 | ⮃ | ✓ | ✓ | ✓ | ⮃ | ⮃ |
| Goldberg, 2004 |  | ✓ | ✓ |  | ⮃ |  |
| Haffner, 1995 | ✓ | ✓ | ✓ | ✓ | ⮃ | ⮃ |
| Hunninghake, 1993 | ⮃ | ✓ | ✓ | ! | ⮃ | ! |
| Insull, 2005 | ⮃ | ✓ | ✓ | ✓ | ⮃ | ⮃ |
| Kerzner, 2003 | ⮃ | ✓ | ✓ | ✓ | ⮃ | ⮃ |
| Kollerits, 2016 | ✓ | ✓ | ✓ | ✓ | ⮃ | ⮃ |
| Kostis, 1994 | ! | ✓ | ✓ | ✓ | ! | ! |
| Lepre, 1999 | ⮃ | ✓ | ✓ | ✓ | ⮃ | ⮃ |
| Melani, 2003 | ✓ | ✓ | ✓ | ✓ | ⮃ | ⮃ |
| Min, 2013 | ✓ | ✓ | ✓ | ✓ | ⮃ | ⮃ |
| Mishra, 2005 | ⮃ | ✓ | ✓ | ✓ | ⮃ | ⮃ |
| Nawrocki, 1995 | ✓ | ✓ | ✓ | ✓ | ⮃ | ⮃ |
| Nestel, 2013 | ⮃ | ✓ | ✓ | ⮃ | ⮃ | ⮃ |
| Nielsen, 1993 | ⮃ | ✓ | ✓ | ! | ⮃ | ! |
| Notarbartolo, 1995 | ⮃ | ✓ | ✓ | ✓ | ⮃ | ⮃ |
| Saltissi, 2002 | ! | ✓ | ✓ | ! | ⮃ | ! |
| Schanberg, 2012 | ⮃ | ✓ | ✓ | ✓ | ✓ | ⮃ |
| Schrott, 1995 | ⮃ | ✓ | ✓ | ✓ | ⮃ | ⮃ |
| Stein, 2000 | ⮃ | ✓ | ✓ | ✓ | ⮃ | ⮃ |
| Tsimikas, 2004 | ⮃ | ✓ | ✓ | ⮃ | ⮃ | ⮃ |
| Wiegman, 2004 | ✓ | ✓ | ✓ | ✓ | ⮃ | ⮃ |
| Winkler, 2004 | ⮃ | ✓ | ✓ | ⮃ | ⮃ | ⮃ |
| Zambon, 1994 | ⮃ | ✓ | ✓ | ✓ | ⮃ | ⮃ |
